# Analytical investigation of metal distribution from e-cigarette aerosols to lung deposition using multi-platform mass spectrometry

**DOI:** 10.1007/s00216-026-06487-1

**Published:** 2026-04-16

**Authors:** Jack McGrath, Oliver Royle, Andrew Thorpe, Janice Irene McCauley, Maiken Ueland, Irina Kabakova, Hui Chen, David Clases, Brian G. Oliver, Dayanne Mozaner Bordin

**Affiliations:** 1 School of Mathematical and Physical Sciences, University of Technology Sydney, Ultimo, Australia; 2 School of Life Sciences, Faculty of Science, University of Technology Sydney, Ultimo, Australia; 3 NanoMicroLAB, Institute of Chemistry, University of Graz, Graz, Austria; 4 Respiratory Cellular and Molecular Biology, Woolcock Institute of Medical Research, Sydney, Australia

**Keywords:** E-cigarettes, Vape, Lung, Metal speciation, Bioaccumulation

## Abstract

E-cigarettes are promoted as a safer alternative to conventional tobacco, yet emerging evidence indicates they may expose users to harmful metals. While prior research has focused on nicotine and organic constituents, the bioaccumulation and spatial distribution of toxic metals in lung tissue remain poorly understood. In this study, a multi-platform analytical approach combining ICP-MS, GC-ICP-MS, GC-MS, and LA-ICP-MS/MS was employed to characterise the elemental and organic composition of refillable e-cigarette liquid and its corresponding aerosol, and to assess metal accumulation in murine lung tissue following short-term exposure. Balb/c mice (7 weeks, *n* = 8 per group) were exposed to nicotine-containing e-cigarette aerosols (8, 16, or 32 puffs; 30 min, twice daily for 4 days) or ambient air (control). Results showed that multiple toxic metals and organometallic species were detected in both e-liquid and aerosol, including species of Al, Ni, Cu, As, Br, Sn, and Hg. Elemental bioimaging of murine lungs found heterogeneous focal accumulation patterns in lung tissue, with increased Cu, significantly elevated Ni and Pb, and significantly reduced pulmonary Fe concentrations (*p* ≤ 0.005). Metal distribution patterns were exposure-related but not strictly dose-dependent, indicating a complex deposition and clearance mechanism. This study provides the first evidence of metal-containing (organometallic) species in e-cigarette aerosols and exposure-associated metal accumulation in murine lung tissue. These findings reinforce the need for further mechanistic and longitudinal investigations to assess the health risks associated with chronic vaping exposure.

**Graphical abstract:**

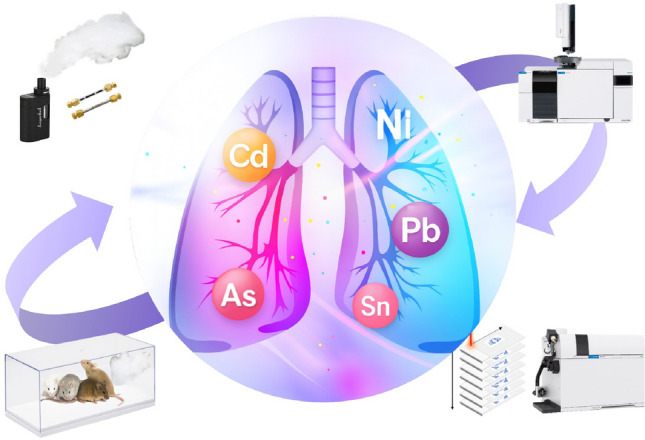

**Supplementary Information:**

The online version contains supplementary material available at 10.1007/s00216-026-06487-1.

## Introduction

Since their introduction in 2004, electronic cigarettes (e-cigarettes, vapes) have been promoted as a safer alternative to traditional tobacco and as an effective tool for smoking cessation aid [1, 2]. These devices deliver inhalable nicotine generated by heating a liquid formulation, a process claimed to avoid the combustion-related by-products such as tar and many carcinogens associated with conventional cigarettes [1, 2]. This perceived reduction in harm, along with misleading marketing campaigns [3, 4], has contributed to the rapid uptake of e-cigarettes globally, particularly among younger demographics. In Australia, for example, e-cigarette use among young adults increased from 5.3% in 2019 to over 21% in 2023, with a similar rise in adolescents [5]. Comparable trends are seen in the USA and the UK, where e-cigarette use among young people has become a significant public health concern [6, 7]. This uptake was partially driven by the availability of unregulated and often illegally imported and low-cost devices, which are sold to children.

The long-term health impacts of e-cigarettes, however, are unclear due to their recent introduction to the market. Nonetheless, inhalation of e-cigarette aerosols has been associated with adverse respiratory outcomes, including chronic obstructive pulmonary disease (COPD), airway inflammation, impaired lung function, and increased oxidative stress, much the same as tobacco [6–8]. Unlike cigarettes, which are a relatively consistent global product, e-cigarette formulations and devices are often manufactured with poor quality control, often involving materials and components with unknown toxicological relevance. Limited regulatory oversight further necessitates systematic investigation of the chemical composition of these products and their potential respiratory impacts [7, 8].

E-cigarette aerosols are complex mixtures containing volatile organic compounds (VOCs), aldehydes, tobacco-specific nitrosamines (TSNAs), and metals such as chromium (Cr), nickel (Ni), copper (Cu), and lead (Pb) [9–11]. These toxicants may originate from device components, including heating elements, or the e-liquids themselves [9]. Previous studies have reported substantial variability in metal and other chemical concentrations across products, reflecting differences in device design, materials, manufacturing quality, and user behaviour [9, 12, 13]. Elevated levels of metals such as Cr, Cu, and Pb have been detected in biological samples (urine, saliva, serum, and blood) of e-cigarette users compared to non-users [14], while prolonged inhalation exposure to Ni and Pb has been associated with pulmonary inflammation, oxidative stress, and structural alterations in lung tissue in experimental and epidemiological studies [15, 16].

Despite this growing body of evidence, the extent to which metals derived from e-cigarette aerosols accumulate within lung tissue, and their spatial distribution following inhalation, remains poorly characterised. Recent investigations indicate that device components composed of Ni-Cr alloy, stainless-steel, and other internal materials can contribute to metal transfer into e-liquids and aerosols during use, with release influenced by device ageing and coil degradation. Metals such as Cr and Ni are primarily associated with heating elements, whereas Pb, Cu, Zn, and antimony (Sb) may originate from non-heating components. The speciation studies demonstrate that metals are present in defined chemical forms, such as Cr predominantly as Cr(III) and Sb as mixed Sb(III)/Sb(V) species, emphasising the importance of integrating speciation with total elemental analysis when evaluating exposure and potential health implications [17]. This information is essential for assessing the long-term health risks, particularly in vulnerable populations, and for informing regulatory policies.

Toxic metal exposure is a recognised global health concern, with some acting as systemic toxicants even at low levels [18, 19]. Accordingly, there is a need for robust analytical characterisation of metals and metal species associated with e-cigarette use. Therefore, this study investigated whether short-term exposure to e-cigarette aerosols leads to metal accumulation in lung tissue, alongside a systematic characterisation of elemental and metal-containing species in the aerosol. It used a murine inhalation model with complementary mass spectrometric techniques, including high-resolution elemental bioimaging, to quantify tissue-associated metals and determine their spatial distribution within the lung.

## Materials and methods

### Chemicals, reagents, and solutions

High-purity single-element standard calibration standards (1000 mg L⁻^1^ for Al, Cr, Fe, Ni, Cu, Zn, As, Br, Sn, Hg, Pb, and Rh) and ultrapure nitric acid were purchased from Choice Analytical (Thornleigh, NSW, Australia). Nicotine, chlorobenzene-d₅ analytical standards, and materials used for gelatine calibration, including HybriWell™ mold, high-purity porcine gelatin, and BT Bio-Rex 70 resin (sodium form), were obtained from Sigma-Aldrich (Thornleigh, NSW, Australia). Organometallic standards, including tetrabutyltin (1000 µg mL^−1^), tetraethyl lead (200 µg mL^−1^), and arsenobetaine (1000 µg mL^−1^) were obtained from Novachem (Heidelberg West, VIC, Australia). Ultrapure water (18.2 MΩ cm) was obtained from a Sartorius 611 arium® pro system and used for all preparations and dilutions. High-purity organic solvents methanol, acetonitrile, xylene, and ethanol were purchased from ChemSupply (Gillman, SA, Australia).

### Instrumentation, experimental parameters, and sample preparation

A multi-platform analytical approach was employed to characterise the elemental and organic composition of e-cigarette liquid and to assess the presence of metal-containing species in the corresponding aerosol, as well as to evaluate metal accumulation in lung tissue following the exposure protocol. Instrumental parameters were systematically optimised prior to analysis. Linearity for quantitatively determined analytes and elements was assessed by calculation of the Pearson correlation coefficient (*R*^2^ ≥ 0.99 in all cases). Calibration ranges and regression parameters are provided in the Supplementary Material (Figures S1 to S5). Elements were selected based on toxicological relevance and reported occurrence in vaping products, particularly those associated with coil alloys, solder joints, and other device components [20–22]. Initial semi-quantitative screening across analytical platforms was used to refine element selection. For TD-GC-ICP-MS, analytes were prioritised based on detection in bulk metal analysis and their capacity to form volatile or semi-volatile species under the applied conditions. LA-ICP-MS screening of lung tissue was used to confirm the presence of exposure-related elements in situ and to guide targeted imaging. To preserve dwell time, counting statistics, and spatial fidelity, the number of isotopes included in each LA-ICP-MS imaging run was deliberately limited. This tiered strategy ensured that all elements subjected to detailed quantitative and spatial analysis were both analytically robust and biologically relevant within the exposure model.

*GC-MS analyses of nicotine and other volatile compounds* in e-liquid were performed using a TRACE 1310 gas chromatograph coupled to a single-quadrupole mass spectrometer with an AI 1310 autosampler (Thermo Fisher Scientific, Australia). Data acquisition and processing were conducted using Chromeleon Chromatography Data System software (version 7.2). The mass spectrometer was tuned and mass calibrated according to the manufacturer’s recommendations prior to analysis. Helium (99.999% purity, BOC, North Ryde, NSW, Australia) was used as the carrier gas. Samples were diluted 1:2000 in methanol prior to analysis.

A 1-µL aliquot was injected, and analyses were performed in full-scan and selected ion monitoring (SIM) modes. Full-scan acquisition was used for untargeted identification of VOCs, while SIM mode was used for targeted nicotine quantification. External calibration standards were prepared in methanol over the range 5–50 μg g^−1^. Solvent blanks were analysed periodically to monitor carryover and contamination. All samples were analysed in triplicate under conditions shown in Table 1. Volatile compound identification was performed by comparison with the NIST mass spectral library, with acceptance criteria of match factors ≥ 700, supported by evaluation of molecular ions, diagnostic fragments, ion ratios, and chromatographic resolution [25]. *TD-GC-ICP-MS elemental speciation analysis* was performed using an 8890 gas chromatograph coupled to a 7900 ICP-MS (Agilent Technologies, Santa Clara, CA, USA) via a dedicated GC-ICP-MS interface comprising a temperature-controlled transfer line and a heated stainless-steel injector tip to ensure efficient analyte transfer. Data acquisition and processing were carried out using MassHunter software (version 4.6, C.01.06). The general performance of the ICP-MS system was monitored using a multi-element tuning solution (Li, Y, Tl, Ce, and Ba; 1 ng mL⁻^1^) in stand-alone mode. Detector pulse/analogue (P/A) calibration was performed using a multi-element standard supplied by the manufacturer. During GC-ICP-MS operation, a Xe-He gas mixture was introduced into the plasma, and the ^12^⁶Xe signal was monitored to optimise plasma stability and analytical sensitivity.

**Table 1:** Operating conditions for the chromatographic separation instruments

Instrument	Settings
**Thermo 1310 GC-MS**	
Injection mode	Splitless
Injection volume	1 μL
GC inlet temperature	280 °C
Column phase	(1) Agilent DB-5MS UI (25 m, 0.25 mm, 0.25 μm) and (2) Agilent DB-WAXETR (30 m, 0.320 mm, 1 µm)
He gas flow	1.2 mL/min
Oven temperature program	(1) 50 °C hold for 2 min → ramp at 25 °C min^−1^ to 150 °C → ramp at 3 °C min^−1^ to 200 °C → ramp at 8°Cmin^−1^ to 300 °C → hold at 300 °C for 7 min (2) 90 °C hold for 2 min → ramp at 15 °C min^−1^ to 150 °C → ramp at 10 °C min^−1^ to 210 °C → ramp at 8°Cmin^−1^ to 300 °C → hold at 300 °C for 7 min
MS transfer line and ion source temperature	250 °C, 230 °C
Dwell time	30 ms
Scan range mass-to-charge ratio (m/z)	40–550 Da
SIM ions	162, 133, 84
**TD-GC**	
Thermal desorber	Tubes heated to 300 °C for 4 min; cold trap TenaxTA/Carbograph 1TD at −10 °C; trap desorbed at 300 °C for 3 min with split flow of 20 mL min^−1^
Injection mode	Splitless
Injection volume	1 μL
GC inlet temperature	250 °C
Column phase	Agilent HP-5 (30 m, 0.32 mm, 0.25 μm)
He gas flow	2 mL/min
Oven temperature program	50 °C hold for 1 min → ramp at 30 °C min^−1^ to 150 °C → ramp at 3 °C min^−1^ to 290 °C → hold at 290 °C for 3 min
ICP transfer line	280 °C
**7900 ICP-MS**	
RF power	700 W
Extract lens 1 and 2	−120.0 V, −9.0 V
Omega bias, lens and RF	−90 V, 10.0 V, 1.40 V
Deflect	2.0 V
He collision gas	3.0 L min^−1^
Isotopes	^7^Li, ^27^Al, ^35^Cl, ^52, 53^Cr, ^54, 55, 56^Fe, ^60^Ni, ^63, 65^Cu, ^66^Zn,^75^As, ^79^Br, ^111, 114^Cd, ^118, 119, 120^Sn, ^201, 202^Hg, ^206, 207, 208^Pb
**LECO GC×GC-TOF-MS**	
Primary column	Rxi®−624Sil MS (30 m × 0.25 mm × 1.40 μm)
Secondary column	Stabilwax® (2 m × 0.25 mm × 0.5 μm)
Carrier gas	He, 1.0 mL min⁻^1^
Primary oven program	35 °C (5 min) → 5 °C min⁻^1^ to 240 °C → hold 5 min
Secondary oven and modulator offset	15 °C, 5 °C
Modulation period	5 s
GC transfer line	250 °C
TOF-MS ionisation mode	Electron ionisation (EI)
Ionisation energy	− 70 eV
Ion source temperature	200 °C
Mass range (m/z)	29–450 Da
Acquisition rate	100 spectra s⁻^1^
Detector voltage offset	200 V

E-cigarette aerosol collection was performed following an approach adapted from established sorbent-tube sampling workflows for VOC analysis in air and aerosol matrices [26–28]. Aerosol was generated under controlled puffing conditions; briefly, a 100-mL aliquot was drawn using an EasyVOC pump onto Tenax® SS thermal desorption tubes over a 5-s collection period. Following collection, analytes were thermally desorbed using a Markes UNITY 2 thermal desorber coupled to a Series 2 ULTRA multi-tube autosampler (Markes International Ltd) and transferred directly into the GC-ICP-MS system for analysis. Blank thermal desorption tubes were analysed before and after sample runs to assess background contamination and monitor potential carryover.

Detection of metal-containing species was performed using chromatographic isotope-specific detection. Retention time alignment approaches, supported by the analysis of selected standards under comparable conditions, were used to assist in the tentative assignment of metal-associated species. Target isotopes were monitored to confirm species identity and to minimise potential spectral interferences from polyatomic ions or co-eluting organometallic species, with isotopic ratios evaluated where applicable. Aerosol TD-GC-ICP-MS data were interpreted qualitatively to confirm the presence of metal-containing species. The sample was analysed in triplicate under the instrumental conditions detailed in Table 1.

*TD-GC × GC-TOF-MS analysis for tentative identification of organic and metal-associated compounds* in aerosol samples was performed using a Pegasus 4D BT GC×GC-TOF-MS system (LECO, Castle Hill, NSW, Australia). Data acquisition and processing were conducted using ChromaTOF® software. The mass spectrometer was tuned and mass calibrated according to the manufacturer’s recommendations prior to analysis. Helium (99.999% purity, BOC, North Ryde, NSW, Australia) was used as the carrier gas.

Aerosol sample collection and TD settings were consistent with those applied for TD-GC-ICP-MS analysis. An internal standard (chlorobenzene-d₅) was introduced prior to analysis to support data normalisation and quality control. TD settings were consistent with those applied for TD-GC-ICP-MS analysis. Blank sorbent tubes were analysed to monitor background contamination and carryover [26–28]. All samples were analysed in triplicate under the conditions detailed in Table 1.

Data processing in ChromaTOF® was performed for peak detection and deconvolution using a signal-to-noise ratio threshold of 150 and a baseline offset of 0.8. Peak widths of 30 s and 15 s were applied for the first and second chromatographic dimensions, respectively. Compound identification was performed by comparison with the NIST mass spectral library, with a minimum similarity match of 80%, supported by evaluation of spectral fragmentation patterns and two-dimensional retention time alignment. As GC×GC-TOF-MS does not provide element-specific detection, assignment of metal-associated species is considered tentative and based on indirect evidence. Data were interpreted in conjunction with TD-GC-ICP-MS results to enable correlation between molecular composition and element-specific signals within the aerosol samples.

*A 7700 ICP-MS* (Agilent Technologies, Santa Clara, CA, USA) was used to perform total metal quantification of e-cigarette liquids. Data acquisition and processing were carried out using MassHunter software (version 4.6, C.01.06). The instrument was tuned daily using a multi-element tuning solution (Li, Y, Ce and Tl; Agilent Technologies) to optimise sensitivity and mass calibration while minimising oxide and doubly charged ion formation. E-liquid samples were prepared using an acid digestion protocol adapted from previously validated analytical workflows for metal determination in vaping products [20, 21, 29]. Briefly, a 45-μL aliquot of e-liquid was digested with 200 μL of concentrated (69%) ultrapure nitric acid at 80 °C for 1 h using a digital dry block heater (Ratek Instruments, Australia), then diluted to a final volume of 10 mL with Milli-Q water. External calibration standards were prepared over the concentration range 0.01 ng g⁻^1^ to 0.1 µg g⁻^1^. Rhodium (Rh) at 0.05 µg g⁻^1^ was used as an internal standard for all calibration standards and samples. Samples were analysed in triplicate under the instrumental conditions detailed in Table 2.

**Table 2:** Operating conditions for the ICP-MS, ICP-MS/MS, and laser ablation

Instrument	Settings
**Agilent 7700 ICP-MS**	
RF power	1550 W
Nebulizer gas	1.65 L min^−1^
Extract lenses 1 and 2	10.0 V, −165.0 V
Omega bias, lens, and RF	−75.0 V, 8.4 V, 1.56 V
Deflect	−2.4 V
H2 collision gas	3.2 L min^−1^
Isotopes	^7^Li, ^9^Be, ^27^Al, ^52^Cr, ^55^Mn, ^56^Fe, ^60^Ni, ^63^Cu, ^66^Zn, ^75^As,^79^Br,^103^Rh, ^111^Cd, ^118^Sn, ^121^Sb, ^182^W, ^201^Hg, ^208^Pb
**CETAC LSX-213 G2+ laser**	
Laser wavelength	213 nm
Pulse energy, repetition rate	20% (1.64 J cm^−2^), 20 Hz
Spot size and scan speed	30 µm, 60 µm s^−1^
Laser MFC 1 and 2 He flow	0.35 (cell gas), 0.1 ml min^−1^ (ablation cup)
**Agilent 8900 ICP-MS/MS**	
RF power	1550 W
Nebulizer gas	1.08 L min^−1^
Extract lens 1 and 2	3.5 V, −170.0 V
Omega bias, lens, and RF	−195.0 V, 11.2 V, 1.80 V
Deflect	−5.6 V
He collision gas	3.2 L min^−1^
Isotopes	^52^Cr, ^56^Fe, ^60^Ni, ^63^Cu, ^66^Zn, ^75^As, ^118^Sn, ^201^Hg, ^208^Pb

*LA-ICP-MS/MS experiments of lung tissues* were conducted using a CETAC LSX-213 G2+ laser ablation system (Teledyne Photon Machines, Bozeman, MT, USA), coupled to an 8900 ICP-MS/MS (Agilent Technologies, Santa Clara, CA, USA). Helium (99.999% purity; BOC, North Ryde, NSW, Australia) was used as the carrier gas. The ICP-MS/MS instrument was tuned for maximum sensitivity before each measurement using NIST SRM 612 glass to maximise sensitivity. Ce/CeO isotopes ratios were monitored to confirm minimal oxide formation and the absence of significant polyatomic interferences. The total integration time sweep was 0.25 s, producing square image voxels that maintained relative specimen image dimensions.

Prior to analysis, paraffin-embedded lung sections were dewaxed with xylene, followed by methanol. Quantification was achieved using gelatin-based calibration standards, prepared in Tris-HCl buffer (pH 7.4) containing 10 mM EDTA and 1% (w/w) polyethylene glycol (M_n_ 400) following established protocols [23]. Calibration curves were constructed by plotting average signal intensities of each element against concentration (µg g^−1^) using five individual ablation lines of each standard. Pew2 software (version 1.7.0) was used for image reconstruction [24]. Elemental concentrations in gelatine standards were verified in triplicate by solution nebulisation ICP-MS/MS following acid digestion [23]. Using the resulting linear regressions, each LA-ICP-MS voxel was converted into concentrations. Typical instrument parameters are outlined in Table 2.

### Animal e-cigarette exposure protocol and ethics statement

All murine experiments were approved by the Animal Care and Ethics Committee at the University of Technology Sydney (approval #ETH18-3179) and conducted in accordance with the Australian NHMRC Guide for the Care and Use of Laboratory Animals. Balb/c mice (7 weeks of age, Animal Resource Centre, WA, Australia) were housed in individually ventilated mouse cages with environmental enrichment at 20 ± 2 °C under a 12-h light/dark cycle (lights on at 06:00 h) with ad libitum access to standard laboratory chow and water. They were allowed a week to acclimatise to the new environment. Balb/c mice were selected for this study as they are one of the most susceptible strains to cigarette smoke–induced respiratory inflammation and airway remodelling, showing reproducible pulmonary responses analogous to those in human smokers [30–34].

The experimental design followed protocols previously described for this strain [35]. Briefly, mice were randomly assigned into four groups (*n* = 8 per group): control (ambient air), 8, 16, or 32 puffs exposure groups. To assess the effects of nicotine-containing e-cigarette aerosols, mice were exposed for 30 min (InExposure Chamber, Scirep, QC Canada) twice daily for consecutive 4 days according to previously published protocols [30, 33, 35]. The final exposure occurred at 15:00 h, and mice were sacrificed after, between 08:00 and 10:00 h the following morning under deep anaesthesia using isoflurane (2%). Lungs were excised, fixed in 4% paraformaldehyde, paraffin-embedded, and sectioned (5 µm).

Exposures were performed using a KangerTech CUPTI device (Vaper Empire, VIC, Australia), a refillable adjustable-wattage model with a fixed 5 V input and replaceable 18650 Li-ion battery. The device supports Ni200, titanium (Ti), stainless-steel (SS), and nickel-chromium (NiCr) coils, as confirmed by manufacturer specifications. The operating temperature range (100–300 °C) was based on manufacturer information and user manual. Each cartridge comprised a Pyrex glass tank, polyoxymethylene drip tip, and a flat NiCr heating element (1.5 Ω resistance). A refillable device was used to enable standardised and reproducible aerosol generation with controlled e-liquid composition and operating conditions, which is required for consistent dose delivery in animal exposure protocols. E-liquid (KangerTech, tobacco flavour, 18 mg·mL⁻^1^ nicotine) contained a 50:50 mixture of propylene glycol (PG) and vegetable glycerine (VG). The nicotine used was free-base nicotine. Aliquots of e-liquid were analysed after device activation.

### Statistical analysis

Data acquisition and primary processing were performed using the instrument manufacturer’s software. Statistical analyses for quantitative datasets were conducted using the Data Analysis ToolPak in Microsoft Excel. Instrument responses were converted to concentrations using calibration curves, with dilution factors applied as required.

For quantitative ICP-MS and chromatographic analyses, descriptive statistics (mean, standard deviation, and coefficient of variation) were used to evaluate analytical precision and recovery. Limits of detection (LOD) and quantification (LOQ) were calculated in accordance with analytical guidelines.

LA-ICP-MS/MS elemental imaging data were visualised and processed using Pew^2^ software. Prior to analysis, elemental images were treated using a rolling median noise filter (window size 5, *k* = 3.0). Background signals arising from glass substrates were segmented and removed using a k-means clustering algorithm (*k* = 3, *t* = 1). Following background correction, signal intensity histograms were generated and mean values were calculated for comparative analysis between lung tissue groups.

Statistical comparisons of lung tissue elemental data were performed using GraphPad Prism version 9.4.0 (GraphPad Software, San Diego, CA, USA). For non-parametric data with heterogeneous distributions, the Kruskal-Wallis test was applied, followed by Dunn’s multiple comparisons test to compare mean ranks across groups. Data visualisation and additional image handling were performed using MATLAB (MathWorks, Natick, MA, USA). For figure preparation, Pew^2^ image data were exported to ParaView (version 5.13.3) to generate high-resolution images. Statistical significance was defined as *p* ≤ 0.05.

## Results and discussion

With the perceived benefits, increasing uptake and ease of use of e-cigarettes by younger generations, particularly among adolescents, the potential health risks require immediate investigation. The purpose of this research was to evaluate whether exposure to e-cigarette aerosols leads to metal accumulation in lung tissue following short-term inhalation. To enable this assessment, a systematic chemical characterisation of refillable e-cigarette liquid and its corresponding aerosols was first performed to identify the elemental and metal-containing species present prior to exposure. This was achieved using a multi-platform analytical approach integrating GC-MS, TD-GC×GC-TOF-MS, TD-GC-ICP-MS, ICP-MS, and LA-ICP-MS/MS, providing complementary information across liquid, aerosol, and tissue matrices.

### GC-MS and TD-GC×GC-TOF-MS characterisation of e-cigarette

Nicotine concentration in the e-liquid was measured at 19.50 ± 0.24 mg mL⁻^1^, consistent with the concentration specified by the product vendor and within regulatory limits for vaping products [38, 39]. GC-MS analysis found a chemically complex mixture of volatile and semi-volatile organic compounds in the e-cigarette aerosol, with compound profiles dependent on column polarity. Using the polar column (WAXETR), short-chain organic acids (acetic, formic, and propanoic acids), humectants (propylene glycol, glycerine, and dipropylene glycol), nicotine, and flavour-related compounds such as ethyl maltol and 2-pyrrolidone were identified with high spectral match confidence (≥ 800). Further analysis using the non-polar DB-5 column indicated the presence of glycerine, nicotine, diglycerol, cotinine, phenolic compounds, and long-chain fatty acids. TD-GC×GC-TOF-MS aerosol analysis enabled the detection of additional volatile and semi-volatile organic compounds, including aldehydes, furans, aromatic hydrocarbons, and glycol-derived species consistent with known thermal degradation products of nicotine, propylene glycol, and glycerol-based e-liquids [40–42].

These findings are consistent with previous reports showing that e-cigarette liquids and aerosols contain a wide range of constituents, including nicotine, solvent carriers (propylene glycol and glycerol), tobacco-specific nitrosamines, aldehydes, metals, volatile organic compounds, phenolic compounds, polycyclic aromatic hydrocarbons (PAHs), flavourings, tobacco alkaloids, and other potentially harmful constituents, many of which are known to be toxic, carcinogenic, or associated with respiratory and cardiovascular effects [40, 41]. Previous studies have also demonstrated that the chemical constituents of e-cigarette aerosols are influenced by device parameters and heating conditions. For example, Resende et al. [42] analysed 34 commercial e-liquids and their aerosols using headspace solid-phase microextraction (HS-SPME) and comprehensive two-dimensional GC×GC-MS, identifying 126 compounds, along with the formation of potentially harmful constituents such as furfural, glycidol, and diacetyl during heating. As a result, there is a need to further understand the chemical composition and potential toxicological effects of e-cigarette aerosols on users.

While no metal-containing species were detected under the applied GC-MS and TD-GC×GC-TOF-MS conditions, this outcome likely reflects the matrix complexity and physicochemical constraints of the technique rather than evidence of their absence. Both techniques require analytes to be sufficiently volatile and thermally stable to pass through the chromatographic system. Several metal-containing species that may be present in aerosols are non-volatile or thermally labile, preventing chromatographic transfer and molecular detection under electron ionisation conditions. Accordingly, these techniques were primarily able to characterise the volatile and semi-volatile organic fraction of the sample [43, 44].

In contrast, TD-GC-ICP-MS enables elemental detection following chromatographic separation. Eluting species are introduced into the ICP, where they are atomised and converted to elemental ions prior to mass analysis. Detection is therefore independent of molecular ion formation and EI fragmentation behaviour. This allows selective detection of metal-containing species that are not amenable to conventional molecular analysis and can provide complementary molecular and elemental information for a more comprehensive characterisation of e-cigarette aerosol constituents [45].

### Organometallic speciation of e-cigarette aerosol

**Fig. 1:**
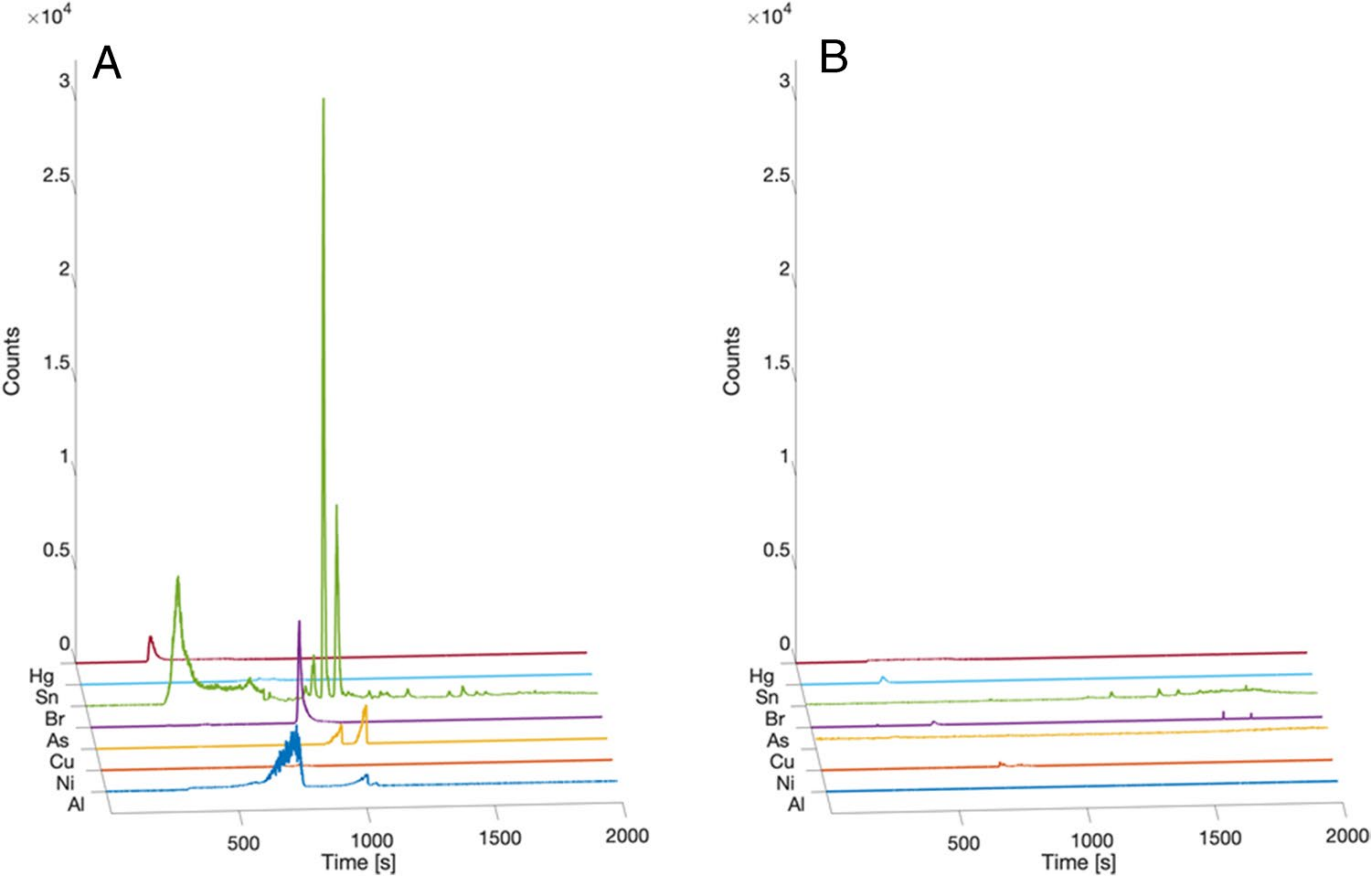
Representative chromatograms of organometallic species detected in e-cigarette aerosol. **A** Chromatographic separation of Al, Ni, Cu, As, Br, Sn, and Hg species in aerosol samples from a Kangertech e-cigarette device (*n* = 3 replicates). **B** Corresponding air blank TD Tenex tube analysis showing the absence of these species. Analyses were performed using TD-GC-ICP-MS

TD-GC-ICP-MS analyses were able to detect multiple organometallic species in the e-cigarette aerosol sample administered to exposed mice. Specifically, species of Al, Ni, Cu, As, Br, Sn, and Hg were detected. The chromatographic profiles demonstrated several distinct peaks corresponding to a number of metal species in aerosol samples, which were absent or negligible in air blank controls, indicating their origin from the e-cigarette device or e-liquid components (Fig. 1 and Figure S1).

As outlined above, attempts to assign molecular identities to these metal species using complementary GC-MS and GC×GC-TOF-MS were not successful. In addition, retention time comparison with available analytical standards analysed under TD-GC-ICP-MS conditions did not yield matching chromatographic features. The absence of both correspondence spectra and retention time alignment likely reflects the inherent chemical complexity, low concentrations, and thermal instability of organometallic species, as well as the limitations of current analytical approaches for their characterisation [46].

TD-GC-ICP-MS provides element-specific detection independent of molecular ionisation, enabling sensitive detection of metal-containing chromatographic entities at trace levels [46]. While the use of direct thermal desorption allows rapid analysis without derivatization or extensive sample preparation, it limits the ability to perform compound-specific speciation required for unambiguous structural or oxidation state identification. As such, detected species are reported as metal-containing chromatographic entities rather than fully characterised molecular compounds. Definitive identification would require further investigation using orthogonal high-resolution or targeted approaches (e.g. GC-Orbitrap or targeted derivatisation strategies), which were beyond the scope of this study [47]. Nevertheless, the reproducible detection of element-specific chromatographic peaks provides clear evidence for the presence of volatile or semi-volatile metal-containing species in the aerosol phase.

The presence of these species is of toxicological relevance, as they can exhibit greater mobility and bioavailability than non-volatile inorganic forms [48–50]. Metals such as mercury, tin, and arsenic are well-recognised environmental pollutants, with organic forms exerting toxicity even at low doses [49]. Their lipophilicity allows them to cross biological membranes more readily, accumulate in tissues, and perturb biological pathways, disrupting endothelial integrity, lipid metabolism, and coagulation, thereby contributing to diseases such as atherosclerosis and cardiovascular disorders [50, 51]. Organotin species, for instance, are potent neurotoxicants that induce inflammation, demyelination, and neuronal cell death [52], while organoarsenic compounds have been linked to genotoxicity and acute toxicity in model systems [53, 54].

The formation of organometallic compounds requires reactive ligands and suitable conditions. It is plausible that high temperatures at the coil interface, coupled with organic solvents in the e-liquid, facilitate organometallic synthesis during vaping [55]. Recent reports of elevated toxic metal emissions from disposable e-cigarettes [17] support our findings that both device hardware and heating processes contribute significantly to toxicant release and prompt the need for more stringent regulation of device composition and performance [56, 57].

### Total metal quantification of e-cigarette used in murine exposure protocol

The e-cigarette liquid used in the exposure protocol was analysed using ICP-MS. The analysis showed elevated concentrations of several metals, including toxicologically relevant ones. These findings are summarised in Table 3.

**Table 3:** Total metal concentrations in the e-cigarette liquid used in mice

Element	Concentration in µg g^−1^ (± SD)
^27^Al	536 ± 5.05
^52^Cr	17.8 ± 5.24
^56^Fe	8.32 ± 0.95
^60^Ni	128 ± 10.76
^63^Cu	138 ± 19.9
^66^Zn	95,514 ± 2.55
^75^As	95.5 ± 12.4
^79^Br	5.12 ± 0.12
^118^Sn	61.9 ± 18.6
^121^Sb	0.74 ± 0.05
^182^W	1.11 ± 0.01
^202^Hg	17.8 ± 0.02
^208^Pb	8.54 ± 0.12

Zn was the predominant element (95,514 ± 2.55 µg g⁻^1^), exceeding the next most abundant metal (Al, 536 ± 5.05 µg g⁻^1^) by approximately two orders of magnitude. Cu (138 ± 19.9 µg g⁻^1^) and Ni (128 ± 10.76 µg g⁻^1^) were present at comparable concentrations, followed by As (95.5 ± 12.4 µg g⁻^1^) and Sn (61.9 ± 18.6 µg g⁻^1^). Cr and Hg were detected at identical mean concentrations (17.8 µg g⁻^1^), while Pb (8.54 ± 0.12 µg g⁻^1^), W (1.11 ± 0.01 µg g⁻^1^), and Sb (0.74 ± 0.05 µg g⁻^1^) were quantified at lower levels. The reported standard deviations represent analytical repeatability, as each digested sample was measured in triplicate by ICP-MS. Relative standard deviations were generally below 10%, supporting analytical precision and excluding contamination or instrumental drift as confounding factors.

The presence of metals such as Ni, Cu, Sn, Pb, and Al is consistent with leaching from internal device components, including heating coils, brass connectors, and solder materials, as reported in previous studies of e-cigarette liquids [12, 36, 37]. In contrast, elements such as As, Br, and Hg are not typical constituents of device alloys and have been previously attributed to trace contamination of e-liquid constituents, including propylene glycol, glycerol, flavouring additives, or nicotine extracts [20, 29, 60].

### Regulatory context and benchmark comparison

Metal-specific concentration limits for e-liquids or e-cigarette aerosols are not defined under current regulatory frameworks (EU Tobacco Products, US FDA, and WHO), which primarily address nicotine content, ingredient disclosure, and general product safety [61–63]. In the absence of product-specific standards, toxicological context is drawn from established inhalation benchmarks and pharmaceutical inhalation impurity limits guidelines.

Occupational exposure limits are intended for airborne workplace exposure and are therefore not directly comparable to liquid-phase concentrations; however, they consistently identify key metals detected here (Ni, As, Pb, Hg) as priority inhalation toxicants. For example, NIOSH recommended exposure limits include 0.015 mg m^⁻3^ for Ni compounds, 0.002 mg m^⁻3^ for inorganic As, and 0.050 mg m⁻^3^ for Pb. In Australia, the Hazardous Chemical Information System (HCIS, Safe Work Australia) lists legally enforceable workplace exposure standards for relevant metals and forms (for example, Pb 0.05 mg m^⁻3^, As 0.05 mg m^⁻3^, Cr(VI) 0.05 mg m^⁻3^, Hg vapour 0.003 mg m^⁻3^, Fe 0.23 mg m^⁻3^, plus form-specific limits for Al, Cu, Sn, and W) [64–66].

Additional context can be obtained from the United States Pharmacopeia (USP 232) [67], aligned with ICH Q3D, which defines permitted limits for elemental impurities in pharmaceutical products by route of administration. For inhalation products, the Individual Component Option provides concentration limits (µg g⁻^1^), enabling direct comparison with measured metal levels in the e-liquid. Several elements substantially exceed these limits. For example, As exceeded the limit by 480-fold (95.5 vs 0.2 µg g⁻^1^), Ni (128 vs 0.5 µg g⁻^1^), Cr (17.8 vs 0.3 µg g⁻^1^), Hg (17.8 vs 0.1 µg g⁻^1^), and Pb (8.54 vs 0.5 µg g⁻^1^) exceeded limits by 250-, 60-, 180-, and 17-fold, respectively. Copper (138 vs 3 µg g⁻^1^) and Sn (61.9 vs 6 µg g⁻^1^) were also elevated, whereas antimony (0.74 µg g⁻^1^) remained below its limit (2 µg g⁻^1^).

Although USP 232 limits were developed for pharmaceutical inhalation products rather than e-cigarettes, they represent a conservative, health-based benchmark for inhalation exposure. In contrast, no equivalent limits are defined for several elements detected in this study, including Al, Cu, Zn, Sn, W, Fe, and Br. The absence of formal limits for these metals reflects a lack of toxicological data for inhalation exposure rather than an absence of hazard, highlighting a current regulatory gap for comprehensive metal risk assessment in e-cigarette products [67]. For example, Zn and Cu are essential elements but can induce respiratory toxicity at elevated inhalation doses, while Al, Sn, and W have been associated with pulmonary inflammation and particulate-driven lung effects in occupational settings. The magnitude by which several metals herein exceed these limits reinforces the concern of metal contamination in the tested e-cigarette liquid and the absence of equivalent product-specific standards for e-cigarette emissions [69, 70].

### Elemental bioimaging of murine lung tissue following e-cigarette aerosol exposure

Lung tissue samples from control (0), 8, 16, and 32 puffs exposure groups were analysed for Cr, Fe, Ni, Cu, Zn, Sn, and Pb using LA-ICP-MS.

**Fig. 2:**
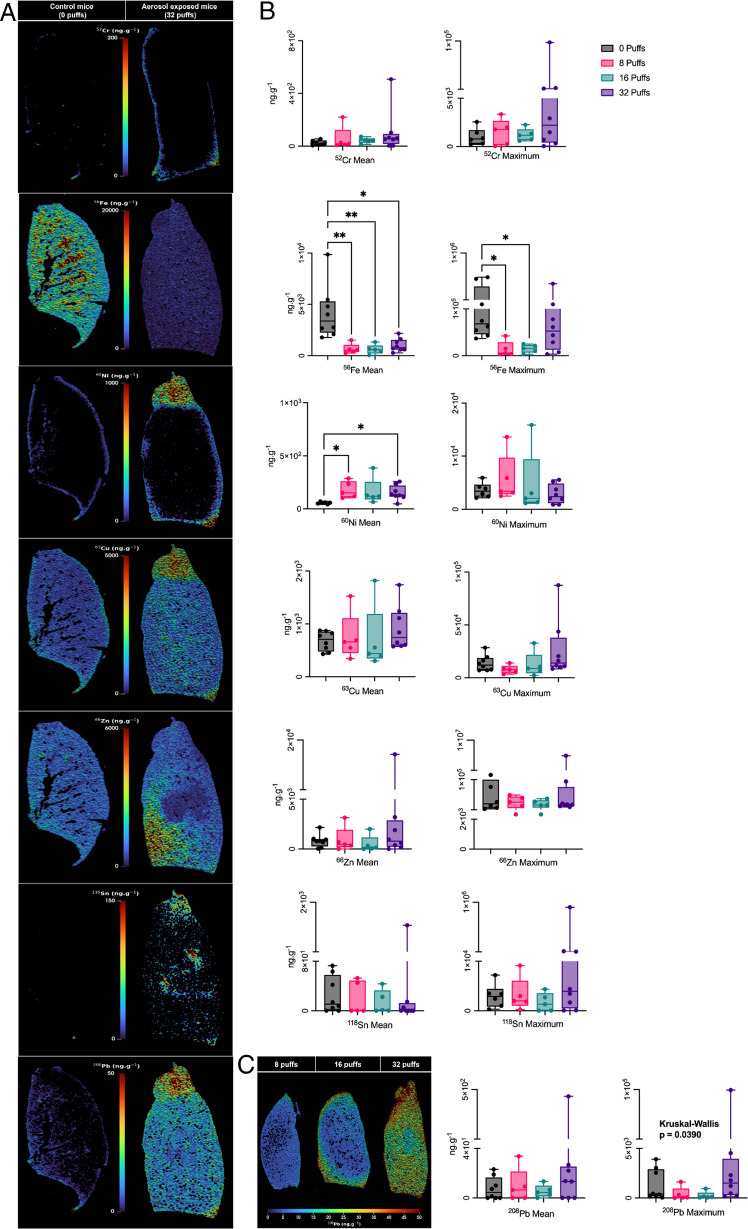
Elemental distribution and quantification of metal accumulation in murine lungs following e-cigarette aerosol exposure. **A** LA-ICP-MS elemental distribution maps of targeted metals in murine lung tissue after a 4-day exposure protocol. Control lung sections (0 puffs) and aerosol-exposed sections (32 puffs) show spatial distribution and relative concentrations (ng g⁻^1^) of Cr, Fe, Ni, Cu, Zn, Sn, and Pb. Notable accumulation is observed in exposed tissues, indicating aerosol-associated metal deposition. **B** Quantitative analysis of lung tissue concentrations of Cr, Fe, Ni, Cu, Zn, Sn, and Pb across exposure groups (0, 8, 16, and 32 puffs). Fe levels significantly increased in both mean (*p* = 0.0009) and maximum (*p* = 0.005) concentrations. Ni showed a significant rise in mean concentration (*p* = 0.0055), while a significant effect of aerosol exposure on maximum Pb concentrations was observed (*p* = 0.0390). Box plots show the full data range (minimum to maximum), with individual data points overlaid. Asterisks indicate statistically significant differences from post hoc testing: *p < 0.05, **p ≤ 0.0055. **C** Representative elemental bioimaging of Pb accumulation in murine lungs following exposure to 8, 16, and 32 puffs administered twice daily over 4 days

Vape exposure significantly reduced Fe concentrations, with both mean (*p* = 0.0009) and maximum (*p* = 0.005) values decreasing across exposure groups, although these patterns were not strictly dose-dependent. Mean Fe levels declined from 4.18 × 10^3^ ng g⁻^1^ in control lungs to 667, 597, and 1.02 × 10^3^ ng g⁻^1^ in the 8, 16, and 32 puff groups, respectively. A representative image of the Fe distribution in both the control (0 puff) and exposed group (32 puffs) is shown in Fig. 2A.

Mean nickel concentrations increased significantly with exposure (*p* = 0.0055), rising from 77.3 ng·g⁻^1^ in controls to 368 ng·g⁻^1^ and 242 ng·g⁻^1^ in the 8 and 32 puff groups, respectively. Copper and lead exhibited similar trends, with maximum concentrations reaching 8.74 × 10^4^ ng·g⁻^1^ for Cu and 9.91 × 10^4^ ng·g⁻^1^ for Pb in the 32 puff group, representing 3.08-fold and 25.3-fold increases compared to control tissue samples. A significant effect of aerosol exposure on maximum Pb concentrations was observed (*p* = 0.0390) (Fig. 2B).

A trend was also observed where the concentrations of Sn were elevated in the 32 puff group, reaching 3.96 × 10^5^ ng·g⁻^1^, corresponding to a 15.4-fold increase compared to controls. Notably, Sn exhibited focal accumulation within the superior lobe, limiting statistical significance in whole-tissue comparisons. This localised deposition pattern was similarly observed for Cr, Ni, and Pb, suggesting region-specific retention of inhaled metals. In the representative images for the elements Cr, Ni, Zn, Sn, and Pb, deposits can be seen in the superior lobe (upper sections) and the pleural cavity of the lung tissue (Fig. 2B).

Representative elemental maps of lead distribution across exposure levels (8, 16, and 32 puffs) are shown in Fig. 2C, illustrating progressive dose-dependent accumulation and spatial heterogeneity.

High concentrations of Cr, Ni, Sn, and Pb were detected in the e-liquid sample, with corresponding organometallic species observed in the aerosol, and elemental bioimaging confirming their deposition in murine lung tissues. LA-ICP-MS images (Fig. 1C) showed region-specific accumulation, with Pb, Ni, and Sn concentrated in the superior lobe and pleural regions, while Zn was predominant in basilar areas. It is plausible that metals are transported to the lung via non-volatile pathways, including association with aerosol droplets, particulate matter, or nanoparticles generated during device heating. The observed spatial variation in metal deposition may likely reflect differences in particle size and physicochemical form. Aerosol deposition is governed by mechanisms such as inertial impaction, sedimentation, and diffusion, which determine regional deposition based on aerodynamic diameter. Larger particles (> 5 µm) deposit in the upper airways, whereas smaller particles (< 2 µm) reach the alveolar region, with intermediate particles depositing in the central airways [68]. In addition, these patterns may not solely reflect differences in initial deposition but also region-specific clearance processes. While particles may deposit throughout the lung, more efficient mucociliary clearance in the upper airways and slower macrophage-mediated clearance in the alveolar region could lead to differential retention and accumulation of metals across lung compartments [69]. It should also be noted that Zn is an endogenous element in lung tissue, and measured levels therefore reflect both baseline physiological concentrations and potential exposure-related contributions, limiting definitive source attribution. These heterogeneous patterns may underlie patchy lung injury and provide guidance for translational studies of vaping-related disease [70].

Of concern is that measurable changes in tissue metal concentrations were observed after only a small number of puffs, indicating that even brief exposure was sufficient to alter metal levels. Increases in Ni, Cu, and Pb were observed following exposure, with Ni levels peaking at intermediate exposure, whereas Fe concentrations decreased most prominently in the low and mid exposure groups. Fe is essential for cellular proliferation, immune defence, and oxidative balance, and its dysregulation has been linked to COPD, cystic fibrosis, and acute respiratory distress syndrome [78]. Therefore, the significant reduction in pulmonary Fe observed here is concerning (Fig. 1A and B). This may reflect true depletion or redistribution to systemic compartments, or enhanced clearance of Fe-containing particles. For example, inhalation of aerosols generated during thermal spray coating, where molten metal is sprayed onto surfaces, has been shown in rats to produce metal-specific effects on the lung. Ni-based wire aerosols were found to cause significant lung injury and inflammation at both 4 and 30 days post-exposure. Zinc cleared rapidly from the lungs within 4 days, whereas Ni, Cr, and Mn persisted at low levels for at least 30 days. However, in the reported study, the assessment of Fe clearance was difficult due to high background levels in the lung, suggesting that observed reductions in pulmonary Fe may reflect redistribution or subtle clearance changes rather than outright depletion [71]. Further, while cigarette smoking has been shown to typically increase lung Fe burden, e-cigarette exposure appeared to reduce Fe, suggesting a distinct perturbation of pulmonary metal homeostasis. Such disruption may impair immune responses and tissue repair. Further research is required to confirm these findings and clarify the underlying mechanisms affecting metal deposition and clearance [72].

Regarding the detection of elevated Ni and Pb in lung tissue after exposure, the toxicological significance is clear. Ni can damage epithelial cells, impair repair, and drive epithelial-mesenchymal transition, raising the risk of fibrosis and COPD [73]. Pb is readily absorbed across the respiratory epithelium and redistributed to blood, bone, and soft tissues, where it contributes to hypertension, vascular dysfunction, and cognitive impairment [74, 75]. The detection of Cu and Sn further raises concern, as Cu dysregulation promotes oxidative stress, while Sn- and As-containing organometallic species are highly bioavailable and known to induce neurotoxicity and genotoxicity [52–54, 76].

Exposure to airborne toxic metals and other environmental toxicants can trigger pulmonary and systemic inflammation, disrupt immune and lung development, and contribute to long-term respiratory dysfunction and disease [77, 78]. Toxic metals can exert negative effects in multiple organ systems, with systemic bioaccumulation depending on exposure route, dose, and duration [79]. The question remains as to whether these metals detected in the lungs remain confined to the respiratory system or enter the circulation to accumulate in distant organs, which requires further investigation. This possibility of redistribution extends the potential health implications of vaping beyond local lung injury. Taken together, the observed patterns of Fe depletion, focal deposition of Pb, and accumulation of Ni, Cu, Sn, and As are particularly concerning, warranting follow-up studies.

These risks are compounded by the high prevalence of e-cigarette use in adolescents, who may be more vulnerable to toxicant effects during critical developmental periods [5–8]. Regulatory gaps, particularly around disposable devices, allow continued exposure to poorly characterised toxicants. Our findings reinforce the urgent need for manufacturing standards, toxic metals testing, and clearer labelling to mitigate these risks.

This study used a refillable KangerTech CUPTI device, which enabled reproducible exposures and aerosol characterisation. Although refillable devices are no longer dominant, recent analyses of popular disposables confirm the presence of the same toxic metals (Al, Cr, Ni, Cu, Sn, Pb). While concentration varies by brand (for example, Ni 1.7–33 µg g^−1^, Pb 64–175 µg g^−1^, and Cu ≥ 350 µg g^−1^), elemental profiles appear broadly consistent across devices. However, disposables have been reported to emit higher levels of Ni, Cu, and Pb, sometimes by orders of magnitude, likely due to coil degradation and lower manufacturing control [17]. Thus, our refillable model may underestimate real-world exposure. For example, a review by Zhao et al. [14] found that metals and metalloids, including Fe, Zn, Ni, Pb, and Sn, are typically present in e-liquids and aerosols and are also detected in human biosamples of e-cigarette users. Notably, most metal and metalloid levels in biosamples of e-cigarette users were similar to or higher than those in conventional cigarette users, and even higher than levels in cigar users. However, there was substantial heterogeneity in metal and metalloid levels depending on sample type, source of e-liquid, and device type.

Concerning the detection of metal-containing species in aerosols by TD-GC-ICP-MS, their presence is noteworthy, with this study providing the first evidence of metal-containing (organometallic) species in e-cigarette aerosols. While detailed structural identification was not achieved within the scope of this work, this finding raises important questions regarding the potential health risks associated with these compounds, particularly given their volatility, mobility, and bioavailability in the lung. This limitation reflects not only the inherent complexity of organometallic compounds, including structural diversity and thermal instability, but also the limited availability of reference spectra and the lack of established standardised analytical protocols, particularly given that TD-GC-ICP-MS is not a commonly or routinely used technique. Although GC-ICP-MS provides sensitive, element-specific detection, it does not yield molecular structural information, and complementary GC-MS analysis did not result in definitive assignments. Resolving the full speciation of these compounds would require orthogonal analytical approaches. High-resolution platforms (e.g. GC-Orbitrap or GC-QTOF) could provide accurate-mass and isotopic pattern information for thermally stable species, supporting molecular formula assignment. For non-volatile or thermally labile compounds, LC-ICP-MS coupled with parallel LC-HRMS would provide complementary elemental and molecular information and represents a strategy for speciation in complex matrices such as e-liquid. In addition, soft ionisation techniques (e.g. APCI-MS) may preserve labile metal-ligand complexes and provide diagnostic fragmentation pathways. This finding prompts further analytical investigation and method development.

Considering both the heterogeneity of metal and metalloid levels in e-cigarette aerosols and the analytical limitations for structural identification, future studies should compare multiple device types, particularly those most commonly used by adolescents, include longer exposure durations, and test diverse e-liquid formulations. Longitudinal studies will be important to evaluate the persistence of lung deposition, systemic redistribution, and chronic respiratory outcomes. Integration of metal speciation with functional readouts, such as lung mechanics, inflammatory markers, and histopathology, will be critical to define mechanisms of injury. In parallel, simplified screening approaches for aerosol-borne metals could support regulation and inform public health strategies. Collectively, these findings provide novel evidence that vaping delivers toxic elements to the lung, disrupts iron homeostasis, and poses risks for pulmonary and systemic health. Given the widespread use of disposable e-cigarettes among adolescents, regulatory oversight of device materials and mandatory metals testing are urgently needed.

## Conclusion

Using a multi-platform analytical approach, we identified toxic metals in e-cigarette aerosols and confirmed their focal deposition in murine lung tissue. Further, the detection of element-specific chromatographic peaks by TD-GC-ICP-MS provides clear evidence for the presence of volatile or semi-volatile metal-containing species in the aerosol phase.

The observed reduction in pulmonary iron, together with accumulation of Pb, Ni, Cu, Sn, and As, indicates disruption of metal homeostasis with potential for both local and systemic toxicity. This is the first study to apply TD-GC-ICP-MS for aerosol speciation in this context, providing insight into vaping-related risks.

Importantly, our preliminary exposure study demonstrates that even short-term vaping can result in measurable metal accumulation in lung tissue. These findings justify further investigation, including studies in human populations, to determine the broader health implications and to inform future toxicological assessments and regulatory strategies.

## Supplementary Information

Below is the link to the electronic supplementary material.

Supplementary file1 (DOCX 32.8 KB)

## Data Availability

Additional data will be made available on request.

## Declarations

### AI use statement

Generative AI tools were used to assist with language editing of the manuscript only. All content was reviewed and verified by the authors.
