# Supplementary material for: Analytical investigation of metal distribution from e-cigarette aerosols to lung deposition using multi-platform mass spectrometry
Source: Anal Bioanal Chem. 2026 Apr 16;418(12):3821–35. doi: 10.1007/s00216-026-06487-1 (PMC13221409; doi:10.1007/s00216-026-06487-1)
Supplement: Supplementary file 1 — Supplementary file1 (DOCX 32.8 KB) [file 216_2026_6487_MOESM1_ESM.docx]

**Supplementary Material**

**Analytical investigation of metal distribution from e-cigarette aerosols to lung deposition using multi-platform mass spectrometry**

Jack McGrath^1^, Oliver Royle^1^, Andrew Thorpe^2^, Janice Irene McCauley^1^, Maiken Ueland^1^, Irina Kabakova^1^, Hui Chen^2^, David Clases^3^, Brian G Oliver^4^, Dayanne Mozaner Bordin^1^

^1^ School of Mathematical and Physical Sciences, University of Technology Sydney, Australia

^2^School of Life Sciences, Faculty of Science, University of Technology Sydney, Australia

^3^NanoMicroLAB, Institute of Chemistry, University of Graz, Graz, Austria

^4^Respiratory Cellular and Molecular Biology, Woolcock Institute of Medical Research, Sydney, Australia

*corresponding author email address: [**Dayanne.Bordin@uts.edu.au**](mailto:Dayanne.Bordin@uts.edu.au)

**Table of contents**

**Table S1** Linear regression parameters and limits of detection for total element quantification by ICP–MS
**Table S2** Linear regression parameters and limit of detection for nicotine quantification by GC–MS **Table S3** Identified volatile and semi-volatile organic compounds in e-liquid by GC–MS using Waxetr and DB-5 columns
**Table S4** Linear regression parameters and limits of detection for multielement LA–ICP–MS lung tissue analysis

**Table S1.** Linear regression parameters used for total elemental quantification via ICP–MS analysis.

| Element | | r² | | Slope | | Intercept | | Sᵧ/ₓ | | LOD (ng·g⁻¹) | |
| --- | --- | --- | --- | --- | --- | --- | --- | --- | --- | --- | --- |
| ^27^Al | | 0.9968 | | 0.0008 | | 0.0074 | | 0.0034 | | 13.44 | |
| ^52^Cr | | 0.9995 | | 0.0071 | | 0.0039 | | 0.0059 | | 2.74 | |
| ^56^Fe | | 0.9971 | | 0.0079 | | 0.0327 | | 0.0165 | | 6.46 | |
| ^60^Ni | | 0.9995 | | 0.0022 | | 0.0022 | | 0.0024 | | 2.95 | |
| ^63^Cu | | 0.9939 | | 0.0077 | | 0.0017 | | 0.0024 | | 1.01 | |
| ^66^Zn | | 0.9943 | | 0.0006 | | -0.0412 | | 0.0372 | | 199 | |
| ^75^As | | 0.9987 | | 0.0010 | | -0.0001 | | 0.0002 | | 0.54 | |
| ^79^Br | | 0.9967 | | 0.0047 | | -0.0022 | | 0.0036 | | 2.80 | |
| ^118^Sn | | 0.9998 | | 0.0497 | | -0.0001 | | 0.0015 | | 0.10 | |
| ^121^Sb | | 0.9985 | | 0.0409 | | 0.0004 | | 0.0003 | | 0.03 | |
| ^182^W | | 0.9983 | | 0.0853 | | 0.0003 | | 0.0007 | | 0.03 | |
| ^202^Hg | | 0.9936 | | 0.0226 | | -0.0023 | | 0.0036 | | 0.53 | |
| ^208^Pb | | 0.9942 | | 0.0172 | | 0.0002 | | 0.0003 | | 0.05 | |

*Reported parameters include coefficient of determination (r²), slope, intercept, residual standard deviation of the regression (Sᵧ/ₓ), and limit of detection (LOD).

**Table S2.**  Linear regression parameters used for nicotine analysis via GC-MS.

| Compound | r² | Slope | Intercept | Sᵧ/ₓ | LOD (ug·L⁻¹) |
| --- | --- | --- | --- | --- | --- |
| Nicotine | 0.9939 | 91950 | -63802 | 115.95 | 3.90 |

*Reported parameters include coefficient of determination (r²), slope, intercept, residual standard deviation of the regression (Sᵧ/ₓ), and limit of detection (LOD).

**Table S3.** Identified volatile and semi-volatile organic compounds in e-liquid by GC–MS using Waxetr and DB-5 columns.

| **Retention time (min)** | **Compound Name** | | **CAS Number** | **Match Factor** | **Probability Match** |
| --- | --- | --- | --- | --- | --- |
| **WAXETR column** |  | |  |  |  |
| 6.53 | Acetic acid | | 64-19-7 | 899 | 85.3% |
| 7.40 | Formic acid | | 64-18-6 | 935 | 86.6% |
| 7.64 | Propanoic acid | | 79-09-4 | 875 | 89.8% |
| 8.44 | Propylene glycol | | 57-55-6 | 903 | 78.1% |
| 11.76 | Dipropylene glycol | | 110-98-5 | 922 | 91.7% |
| 12.40 | Nicotine | | 54-11-5 | 948 | 94.9% |
| 14.25 | Ethyl maltol | | 4940-11-8 | 912 | 88.7% |
| 14.54 | 2-Pyrrolidone | | 616-45-5 | 939 | 97.6% |
| 18.96 | Glycerine | | 56-81-5 | 933 | 94.1% |
| **DB-5** **column** |  | |  |  |  |
| 5.71 | | Glycerine | 56-81-5 | 945 | 94.5% |
| 6.97 | Nicotine | | 54-11-5 | 948 | 83.1% |
| 8.49 | Diglycerol | | 627-82-7 | 831 | 89.9% |
| 9.88 | Cotinine | | 468-56-6 | 918 | 97.5% |
| 11.30 | Palmitic acid | | 57-10-3 | 917 | 74.5% |
| 12.38 | Stearic acid | | 57-11-4 | 949 | 75.8% |
| 12.55 | Phenol, 4,4'-(1-methylethylidene) bis- | | 88-05-7 | 821 | 73.7% |

**Table S5.** Tentatively identified volatile and semi-volatile organic compounds in e-cigarette aerosol by TD–GC×GC–TOF–MS. Compound identification was based on NIST spectral library matching (similarity ≥800) and two-dimensional retention time alignment.

| **Retention time (s)** | **1st Dimension**  **Time (s)** | **2nd Dimension Time (s)** | **Compound Name** | **CAS Number** | **Match Factor** |
| --- | --- | --- | --- | --- | --- |
| 365 | 365 | 2.100 | Furan, 2,5-dimethyl- | 625-86-5 | 982 |
| 380 | 380 | 2.120 | 2-Butenal, 2-ethenyl- | 20521-42-0 | 980 |
| 685 | 685 | 2.255 | Benzene, 1-ethyl-3-methyl- | 620-14-4 | 865 |
| 840 | 840 | 2.685 | Methyl 2,5,8,11-tetraoxatridecan-13-oate | N/A | 744 |
| 980 | 980 | 2.415 | Tetraethylene glycol diethyl ether | 4353-28-0 | 743 |
| 810 | 810 | 4.290 | 1,3,5-Trioxane | 110-88-3 | 966 |

**Table S5.** Linear regression parameters and limits of detection for multielement LA–ICP–MS lung tissue analysis.

| Element | r² | Slope | Intercept | Sᵧ/ₓ | LOD (ng·g⁻¹) |
| --- | --- | --- | --- | --- | --- |
| ^52^Cr | 0.9965 | 1.0149 | 74.1551 | 10.72 | 31.69 |
| ^56^Fe | 0.9806 | 4.462 | 1043.0525 | 216.81 | 145.77 |
| ^60^Ni | 0.9974 | 0.3671 | 21.0326 | 3.32 | 27.13 |
| ^63^Cu | 0.9965 | 1.0149 | 74.1551 | 10.26 | 30.33 |
| ^66^Zn | 0.9976 | 0.8392 | -4.9208 | 15.20 | 54.36 |
| ^75^As | 0.9929 | 0.4131 | -0.569 | 7.16 | 52.01 |
| ^118^Sn | 0.9779 | 2.2696 | 166.0588 | 5.46 | 7.22 |
| ^202^Hg | 0.9945 | 0.8077 | 19.3886 | 1.90 | 7.06 |
| ^208^Pb | 0.9954 | 4.3068 | 18.731 | 4.77 | 3.32 |

*Reported parameters include coefficient of determination (r²), slope, intercept, residual standard deviation of the regression (Sᵧ/ₓ), and limit of detection (LOD).
